# A single-nucleotide polymorphism in the RAD51 gene modifies breast cancer risk in BRCA2 carriers, but not in BRCA1 carriers or noncarriers

**DOI:** 10.1038/sj.bjc.6601837

**Published:** 2004-04-27

**Authors:** L Kadouri, Z Kote-Jarai, A Hubert, F Durocher, D Abeliovich, B Glaser, T Hamburger, R A Eeles, T Peretz

**Affiliations:** 1 Sharett Institute of Oncology, Hebrew University-Hadassah Medical Center, Jerusalem, Israel; 2 Cancer Research UK Section of Cancer Genetics, The Institute of Cancer Research, Sutton, UK; 3 Cancer Genomics Laboratory, CHUL Research Center, Faculty of Medicine, Laval University, Quebec, Canada; 4 Human Genetic Laboratories, Hebrew University-Hadassah Medical Center, Jerusalem, Israel; 5 Department of Endocrinology and Metabolism, Hebrew University-Hadassah Medical Center, Jerusalem, Israel; 6 The Royal Marsden NHS Trust, Sutton, UK

**Keywords:** BRCA1/2, BC risk, RAD51 gene, single-nucleotide polymorphism

## Abstract

Variation in the penetrance estimates for *BRCA1* and *BRCA2* mutation carriers suggests that other genetic polymorphisms may modify the cancer risk in carriers. The *RAD51* gene, which participates in homologous recombination double-strand breaks (DSB) repair in the same pathway as the *BRCA1* and *BRCA2* gene products, is a candidate for such an effect. A single-nucleotide polymorphism (SNP), *RAD51*-135g → c, in the 5′ untranslated region of the gene has been found to elevate breast cancer (BC) risk among *BRCA2* carriers. We genotyped 309 *BRCA1/2* mutation carriers, of which 280 were of Ashkenazi origin, 166 noncarrier BC patients and 152 women unaffected with BC (a control group), for the *RAD51*-135g → c SNP. Risk analyses were conducted using COX proportional hazard models for the *BRCA1/2* carriers and simple logistic regression analysis for the noncarrier case–control population. BRCA2 carriers were also studied using logistic regression and Kaplan–Meier survival analyses. The estimated BC hazard ratio (HR) for *RAD51*-135c carriers adjusted for origin (Ashkenazi *vs* non-Ashkenazi) was 1.28 (95% CI 0.85–1.90, *P*=0.23) for *BRCA1/2* carriers, and 2.09 (95% CI 1.04–4.18, *P*=0.04) when the analysis was restricted to *BRCA2* carriers. The median BC age was younger in *BCRA2-RAD51*-135c carriers (45 (95% CI 36–54) *vs* 52 years (95% CI 48–56), *P*=0.05). In a logistic regression analysis, the odds ratio (OR) was 5.49 (95% CI 0.5–58.8, *P*=0.163). In noncarrier BC cases, carrying *RAD51*-135c was not associated with BC risk (0.97; 95% CI 0.47–2.00). These results indicate significantly elevated risk for BC in carriers of *BRCA2* mutations who also carry a *RAD51*-135c allele. In *BRCA1* carriers and noncarriers, no effect for this SNP was found.

Carriers of a mutated *BRCA1* or *BRCA2* gene are at an increased risk of breast (BC) and ovarian cancer (OC); however, penetrance estimates differ in various study populations (Easton *et al*, 1995; Struewing *et al*, 1997; Ford *et al*, 1998; Thorlacius *et al*, 1998). Modification of the risk by other genes or environmental factors clustering in families probably explains most of this difference. Genes involved in DNA repair, especially those that interact with the product of the *BRCA1* or *BRCA2* genes, are of particular interest as cancer risk modifiers in *BRCA1/2* mutation carriers.

Both BRCA1 and BRCA2 participate in DNA double-strand break repair through homologous recombination (reviewed in Venkitaraman, 2002). RAD51, a eukaryotic homologue of the bacterial RecA protein, is essential for DSB repair. The three molecules co-localise in mitotic and meiotic cells and in DNA foci induced by irradiation. Phenotypic similarities of brca1-, brca2- and rad51-deficient murine models, as well as gross chromosomal rearrangements such as translocations and deletions, which are a common feature of brca1/2 or rad51 null cells, suggest that they function in a common pathway.

A single-nucleotide polymorphism (SNP), g → c, at position 135 of the untranslated region of the *RAD51* gene has been reported (Levy-Lahad *et al*, 2001; Wang *et al*, 2001). The biological effect of this polymorphism is currently unknown. However, in two studies, an elevated BC risk associated with the RAD51-135c allele was reported in *BRCA2* mutation carriers, but not in *BRCA1* mutation carriers (Levy-Lahad *et al*, 2001; Wang *et al*, 2001). A lower risk for OC in *BRCA2* carriers was also suggested by the larger study (Wang *et al*, 2001). Recently, an opposite effect of reduced BC risk was reported in a population of BRCA1-5382insC carriers who also carry the *RAD51*-135c (Jakubowska *et al*, 2003). This polymorphism was not associated with BC risk in a large case–control study in noncarrier population (Kuschel *et al*, 2002).

In order to confirm these results, we assessed the effect of the *RAD51*-135g → c polymorphism on BC risk in *BRCA1/2* mutation carriers and in noncarrier BC cases, mainly of Ashkenazi origin.

## SUBJECTS AND METHODS

### Study population

Two populations were studied: (1) *BRCA1/2* mutation carriers of Ashkenazi and non-Ashkenazi origin and (2) noncarrier, BC patients and a control group without cancer, of Ashkenazi origin. Blood samples from 309 *BRCA1/2* mutation carriers were collected through two centres: 263 carriers were identified through the oncology department and the cancer genetics clinic in the Hadassah Medical Center in Jerusalem, Israel, and 46 through the cancer genetics clinic at the Royal Marsden NHS Trust, London, UK. Cases were tested on the basis of a family history of breast and/or OC or on the basis of their Ashkenazi origin. All but one of the cases from Jerusalem were carriers of one of the three Ashkenazi founder mutations (145 cases: 185delAG in *BRCA1*, 35 cases: 5382insC in *BRCA1* and 82 cases: 6174delT in *BRCA2*). The UK carriers included 17 carriers of Ashkenazi founder mutations (one individual carried both a 185delAG and 6174delT) and 29 other mutations (22 in *BRCA1*; 7 in *BRCA2*). Of the 309 carriers, 177 were affected with BC, 40 with OC and 17 had both cancers. In all, 69 of the mutation carriers were unaffected. Clinical data were unavailable for six of the carriers.

A total of 166 ‘noncarrier’ Ashkenazi Jewish BC patients were ascertained through the oncology department in Hadassah over the period of 1994–98. They were enrolled according to the same protocol as the *BRCA1/2* carriers, but were found to be negative for carrying any of the three Ashkenazi founder *BRCA1/2* mutations. In all, 24 (14%) of the patients were diagnosed below age 40 years, 54 (33%) were aged 40–49 years and 88 (53%) were aged 50+ years. Where data were available (111 noncarrier cases), 71 (64%) reported a positive family history of BC. Thus, this series has a higher frequency of early-onset cases and cases with a family history than would be expected in the Ashkenazi population overall.

The controls were 152 females who took part in an independent study into the genetics of diabetes. All controls were cancer-free, of Ashkenazi origin and aged above 56 years (mean age 69, range 56–92 years). All participants signed an informed consent approved by the local institutional ethics committee or gave permission for samples to be used in an anonymous way for research purposes.

### Genotyping

Genomic DNA was extracted according to standard protocols, and used as a template for the PCR reaction. An SNP of g → c at position 135 in the untranslated region of the *RAD51* gene was analysed using two methods: the ABI/PE Biosystems Taqman system and a standard PCR and digestion technique. Eight samples including three positive for the *RAD51*-135c allele were genotyped using both methods, the results were 100% concordant. By the ABI/PE Biosystems Taqman method, PCR amplification was carried out in a final volume of 25 *μ*l containing 25 ng genomic DNA, 900 nM of each primer, 200 nM FAM-labelled probe, 200 nM VIC-labelled probe and 12.5 *μ*l 2 × Universal PCR master mix (PE Biosystems) containing optimised buffer components and Rox reference dye. The primer sequences were as follows: forward 5′-gca gcc tcc tct ctc cag c-3′; reverse 5′-gct ggg aac tgc aac tca tct-3′. Probe sequences were 5′-Fam-ccc caa cgc ccc tgg ctt ac -3′ and 5′-Vic-caa cgc ccg tgg ctt acg ct-3′. The PCR amplification cycles were 95°C for 10 min, followed by 40 cycles of 95°C for 15 s and 62°C for 1 min. Levels of FAM and TET fluorescence were determined and allelic discrimination was done using the ABI 7700 Sequence detector. By PCR and digestion (Levy-Lahad *et al*, 2001; Wang *et al*, 2001), a PCR product of 157 bp containing the *RAD51*-135g → c polymorphism was amplified using the primers, forward: 5′-tgggaactgcaactcatctgg-3′ and reverse: 5′-gcgctcctctctccagcag-3, in a mixture containing 1.5 mM MgCl_2_ at an annealing temperature of 53°C. The wild-type allele is digested by *Mva*I, resulting in 86- and 71-bp products. This restriction site is missing in the polymorphic c allele. The PCR products were separated on a 3.0% agarose gel with 2 *μ*l (100 ml)^−1^ of ethidiume bromide.

### Statistical analysis

The effects of *RAD51* genotypes on BC risk in mutation carriers were evaluated using a COX proportional hazards model. Participants were followed up retrospectively from the date of birth to several possible outcomes. The outcome in women affected with BC was recorded as the age at first BC diagnosis. Women unaffected with BC were censored at the date of OC diagnosis, prophylactic surgery, date of last follow-up, or death. Since the distributions of age and disease status, and potentially the genotype distribution, were different in the Ashkenazi and non-Ashkenazi populations, the analyses were adjusted for ethnic origin. Although selection of participants is partly based on outcome, this method of analysis was used previously for risk estimation in carriers (Rebbeck *et al*, 1999; Levy-Lahad *et al*, 2001). In our study (Kadouri *et al*, 2001) on the modifying effect of the androgen receptor in *BRCA* carriers, we compared COX proportional hazards models to a variant of the log rank designed to overcome selection bias by comparison of outcome to expected penetrance according to the literature. Since the estimated risks were close in both methods, in the current paper we have used COX proportional hazard models. The significant analysis by COX proportional hazard models (association in BRCA2 carriers) was also analysed using logistic regression analysis and Kaplan–Meier survival analysis, the event was recorded as first BC diagnosis and unaffected women were censored in the same way as for the COX analysis.

Clinical data were not available for six individuals, and six individuals could not be genotyped, so that in the final analysis 297 mutation carriers were included, 191 BC cases and 39 OC cases (including one woman diagnosed simultaneously with both cancers and therefore counted as affected with both cancers) and 67 unaffected carriers.

The case–control analyses based on the non-carrier BC cases (*n*=155) and controls (*n*=142) were performed using a standard logistic regression approach. All analyses were performed using SPSS.

## RESULTS

The *RAD51*-135c heterozygote frequency was 41 out of 303 (13.5%) in all *BRCA1/2* carriers, 35 out of 276 (12.7%) in the Ashkenazi carrier population and six out of 27 (22.2%) in the British (non-Ashkenazi) population (Table 1

**Table 1:** Frequencies of RAD51-135g/c alleles by disease status in BRCA1/2 mutation carriers

**Clinical presentation**	**All^a^ (*n*=297)**	**BRCA1 (*n*=210)**	**BRCA2 (*n*=86)**
Breast cancer	22/124 (17.2%)	13/78 (16.2%)	9/46 (19.6)
Bilateral breast cancer	3/50 (6%)	3/43 (7%)	0/7 (0%)
Breast and ovarian cancer	2/17^b^ (11.7%)	1/12^c^ (8.3%)	1/5^d^ (20%)
Ovarian cancer	6/39 (11.7%)	6/27 (22.2%)	0/12 (0%)
Unaffected	8/67 (11.9%)	7/50 (14%)	1/16 (6.25%)

a Including one carrier of both 185delAG&6174delT mutations.

b Including three individuals with bilateral BC and OC, of which one has RAD51-135c.

c Including two individuals with bilateral BC and OC, of which one has RAD51-135c.

d Including one individual with bilateral BC and OC.

). No *RAD51*-135c homozygotes were identified in any of the study subjects. The difference in allele frequency between the two populations was not significant. However, to control for this difference, risk analyses were adjusted for origin, although the risk estimates changed very little with adjustment. The frequency of *RAD51*-135c carriers according to clinical presentation in *BRCA1* and *BRCA2* carriers is shown in Table 1.

A similar frequency of *RAD51*-135c heterozygotes was found among BC and unaffected *BRCA1/2* carriers (14.1 and 13.2%, respectively, Table 2

**Table 2:** Frequencies of RAD51-135g → c alleles by BC disease status and BC relative risk (RR) in BRCA1/2 carriers and noncarriers

**Genotype**	**BC−a (*n*=106) no. (%)**	**BC+^b^ (*n*=191) no. (%)**	**Av. age at BC onset (year)^c^**	**Breast cancer HR (95% CI)**	***P*-value**
*Carriers*					
BRCA1/2^d^ (*n*=297)					
*Rad51-135c*	14 (13.2)	27 (14.1)		1.28 (0.85–1.9)	
*wt*^e^	92 (86.8)	164 (85.9)		1	0.23
					
BRCA1 (*n*=210)					
*Rad51-135c*	13 (16.9)	17 (12.8)	36.7	1.03 (0.62–1.72)	
*wt*	64 (83.1)	116 (87.2)	41.9	1	0.09
					
BRCA2 (*n*=86)					
*Rad51-135c*	1 (3.6)	10 (17.2)	45.0	2.09 (1.04–4.18)	
*wt*	27 (96.4)	48 (82.8)	45.9	1	0.04
					
*Noncarriers*	(*n*=155)	(*n*=142)		BC RR (95% CI)	
*Rad51-135c*	16 (10.3)	16 (11.3)		1	
*Wt*	139 (89.7)	126 (88.7)		0.97 (0.47–2.00)	0.94

a BC+: affected with breast cancer.

b BC−: unaffected with breast cancer.

c Average age at breast cancer onset for individuals by RAD52-135 genotype.

d Including one carrier of both 185delAG&6174delT mutations.

e wt: wild-type allele.

). Among *BRCA2* carriers, 10 out of 58 (17.2%) affected with BC had the *RAD51*-135c allele, compared with one out of 28 (3.6%) of the unaffected carriers (OC cases included; Table 2). Frequencies in *BRCA1* carriers were 12.8% (17 out of 133) and 16.9% (13 out of 77) in BC cases and unaffected cases, respectively. The estimated BC hazard ratio (HR) in *BRCA1/2* carriers with the *RAD51*-135c allele (Table 2) was 1.28 (95% CI 0.85–1.90, *P*=0.23). A significant effect was found when the *BRCA2* carriers were analysed separately (2.09, 95% CI 1.04–4.18, *P*=0.04). The average age at BC onset was similar in carriers of both the g and c *RAD51*-135 genotypes (45.0 and 45.9 years; Table 2); however, using Kaplan–Meier survival analysis, the median time to BC development was 45 years (95% CI 36–54) in *BCRA2-RAD51*-135c carriers and 52 years (95% CI 48–56) in the *BRCA2-RAD51*-wt group (*P*^log rank^=0.0271, *P*^breaslow^=0.05). In a logistic regression analysis, the estimated odds ratio (OR) adjusted for origin and age was 5.49 (95%CI 0.5–58.8, *P*=0.163). In *BRCA1* carriers, no effect of the *RAD51*-135c was found (HR of 1.03, 95% CI 0.62–1.72, *P*=0.09). The estimation of *RAD51*-135g → c effect on OC in *BRCA1/2* carriers was limited by small numbers.

No effect for the *RAD51*-135c allele on BC risk was found in the noncarrier case–control analysis. The frequency of *RAD51*-135c allele was 11.3% (16 out of 142) in BC cases and 10.3% (16 out of 155) in controls. The estimated relative risk (RR) for BC was 0.97 (95% CI 0.47–2.00, *P*=0.97, Table 2).

## DISCUSSION

Our study of the modifying effect of the *RAD51*-135c → g polymorphism confirms the findings of two previous studies. We found about a two-fold elevated BC risk among *BRCA2* carriers with the *RAD51*-135c allele using COX proportional hazards models. In Kaplan–Meier survival analysis, the median age at BC development was significantly 7 years younger among *BRCA2-RAD51*-135c, compared with *BRCA2-RAD51*-wt carriers. For comparison, we also report the estimated OR by logistic regression analysis, which was also elevated in *BRCA2-RAD51*-135c carriers; however, this was nonsignificant. The *BRCA2-RAD51*-135c comprised only 11 cases rendering an unstable and nonsignificant OR using this model. In addition, since the unaffected carriers are at a substantial elevated lifetime risk for BC, using a simple case–control analysis does not consider the age at onset in the affected group, as well as the length of follow-up in the yet, unaffected controls. The COX proportional hazard model takes into account the time factor, and therefore maybe more suitable for risk analyses in carriers.

Wang *et al* (2001) reported an odds ratio of BC of 3.2 (95% CI 1.4–4.0) based on a population of 216 *BRCA2* carriers, carrying the *RAD51*-135c; it was also associated with a younger age at BC onset. The estimated HR associated with the *RAD51*-135c allele in a study of 67 Ashkenazi *BRCA2* carriers was 4.1 (*P*=0.07; Levy-Lahad *et al*, 2001). Both studies, in agreement with our results, did not observe an effect of the *RAD51* genotype in *BRCA1* carriers. Wang *et al* also found reduced OC risk of 0.40–0.66 among *BRCA2* and *RAD51*-135c carriers, although this was nonsignificant. We could not estimate the associated OC risk due to small numbers. In noncarriers, no BC risk association was found. Interestingly, an opposite effect of reduced BC risk in *BRCA1*-5382insC carriers who also carry the *RAD51*-135c was recently reported (OR=0.23; 95% CI, 0.07–0.62; *P*=0.0015, Jakubowska *et al*, 2003). This study included individuals of Polish ancestry, with much larger frequency of the RAD51-135c allele (17 and 37% in affected and unaffected, respectively) compared to ours and previously reported studies. The number of *BRCA1*-5382insC carriers in our study was too small for separate analysis. However, if these results are confirmed in additional and larger studies, the *RAD51* is the first modifier gene with opposite effects in *BRCA1* compared in *BRCA2* mutation carriers.

The biological effect of the *RAD51*-135g → c SNP is currently unknown; however, since a similar modifying effect has been seen in three separate studies, it is likely that it is a real risk modifier in *BRCA2* carriers. The BRCA2 has a distal role in DNA repair machinery (Davies *et al*, 2001). It directly interacts with the RAD51 protein through the BRC repeat domain and regulates the formation of RAD51 nucleoprotein filaments, which are essential for DNA repair through homologous recombination. However, the direct function of BRCA1 in DSB repair is less clear, a more proximal role in sensing and regulation of cellular response to DNA damage has been suggested by several recent papers (reviewed by Venkitaraman, 2002). The BRCA1–RAD51 complex contains no more than 2–5% of the cellular content of each molecule (Scully *et al*, 1997). Therefore, it is biologically plausible that the effect of *RAD51* SNP is different in *BRCA2* compared with *BRCA1* mutation carriers, although the biochemical mechanism remains to be determined.

Recently, the role of *CHEK2*, also part of the same pathway of the double-strand DNA break repair machinery, was studied in BC patients. The *CHEK2* 1100^*^delC, a truncating variant that impairs the kinase activity of the protein, is associated with an increase of BC risk in *BRCA1/2*-negative individuals with a family history (The *CHEK2*-Breast Cancer Consortium., 2002; Vahteristo *et al*, 2002). This variant conferred no increased cancer risk among *BRCA1/2* carriers or sporadic cases (The CHEK2-Breast Cancer Consortium., 2002). The authors suggest that the lack of effect in *BRCA1/2* carriers is because these genes are part of the same pathway, which is already nonfunctioning in BRCA1 or BRCA2 null cells. This contradicts our finding of a modifying effect associated with the *RAD51*-135c variant. Several possible reasons may account for this controversy. Impaired function of some but not all the additional proteins in a common pathway may be critical to carcinogenesis before the loss of function of the wild-type *BRCA1/BRCA2* allele, while there is still a functioning BRCA1/BRCA2 protein. Alternatively, the type of genetic alteration may be important, for example, a truncating mutation *vs* a modifying polymorphism. In the case of the 6174delT mutation, which comprises the majority of our BRCA2 carrier population as well as in the two before-mentioned studies, part of the BRC repeat domains is still present. Altered function of the RAD51 protein may affect the RAD51-BRCA2^6174delT^ complex and sequester targets of action and further impair the function of the other wild-type (wt) allele as well. However, a truncated protein as in the case of the 1100^*^delC variant may either allow the other wt protein a normal function that results in a low effect on the phenotype of a *BRCA1/2* mutant, or as has been previously suggested, after the loss of the wt *BRCA2* allele, an additional truncated protein would have no effect. Only elucidating the biological basis of the *RAD51*-135g → c polymorphism can provide answers to this controversy.

In the present study, we have reported a modifying effect for the *RAD51*-135g → c SNP in *BRCA2* carriers, similar to the effect reported in two previous studies. This is the first modifier gene identified in *BRCA2* carriers. The clinical implication of these findings is still limited; however, it hints at differences in molecular mechanisms involved in tumour development in *BRCA1* and *BRCA2* carriers. The study of polymorphisms in other DNA repair genes could further elucidate the mechanism of tumorigenesis in *BRCA1* and *BRCA2* carriers.
